# Interaction of theory and experiment: examples from single molecule studies of nanoparticles

**DOI:** 10.1098/rsta.2009.0261

**Published:** 2010-03-13

**Authors:** Rudolph A. Marcus

**Affiliations:** Noyes Laboratory of Chemical Physics, California Institute of Technology, Pasadena, CA 91125, USA

**Keywords:** single molecule studies, nanoparticles, quantum dots, fluorescence intermittency, reaction–diffusion equation

## Abstract

This article is in part the author's perspective on the revolution that has occurred in theoretical chemistry during the past half-century. In this period much of theoretical chemistry has moved from its initial emphasis on analytic treatments, resulting in equations for physical chemical and chemical phenomena, to the detailed computation of many different systems and processes. In the best sense the old and the new are complementary and their coexistence can benefit both. Experiment too has seen major developments. One of the newer types of experiment is that of single molecule studies. They range from those on small inorganic and organic nanoparticles to large biological species. We illustrate some of the issues that arise, using the topic of ‘quantum dots’ (QDs), and choosing a particular inorganic nanoparticle, CdSe, the most studied of these systems. Its study reflects the problems that arise in experiment and in theories in this field. The complementary nature of the conventional ensemble experiments and the new single molecule experiments is described and is illustrated by trajectories for the two types of experiments. The research in the QD field is both experimentally and theoretically a currently ongoing process, for which the answers are not fully known in spite of the large body of research. The detailed role of surface states is part of the problem. The field continues to yield new and unexpected results. In a sense this part of the article is an interim report that illustrates one analytic approach to the topic and where computer calculations and simulations can be expected to provide added insight.

## 1. Introduction

We have been asked, in this celebratory issue, to give a personal touch to our contributions, and I will try to do that. One main focus of the present paper is on the evolving field of the study of single molecule inorganic and organic nanoparticles, in particular their intermittent fluorescence and what we learn from it. The applications of the nanoparticles, real or potential, are wide, ranging from sensing in biology to their use in solar energy conversion. Our focus is on the inner workings of the phenomena.

Theory in the chemical sciences, which also includes its use in biochemistry, biophysics, and any other discipline it can embrace, has undergone a revolution in the past half-century. I and many others in the Royal Society have been a witness to it, and I will write about it as well. The work on the nanoparticles will serve, albeit in a small way (no pun intended), to illustrate some of the analytic theory/computational interface.

## 2. A little history of theory in chemistry

Going back to the earlier days of theory in chemistry, for example to the beginning of the 19th century and Dalton's time, or even earlier, would take us too far afield. Instead, we recall the situation in theoretical chemistry in the first half or so of the 20th century. Research in theoretical chemistry was rich in names, names related to equations on a broad range of phenomena, such as Lennard-Jones, Pauling, Eyring, Debye, Debye–Hueckel, Evans and Polanyi, Gibbs, Boltzmann, Maxwell, Helmholtz, Arrhenius, Onsager, Kramers, Wigner, Feymann, Rice, Bohr, Coulson, Kirkwood, Hammett, Woodward–Hoffmann, and the list goes on. Their equations were kings or queens in the theoretical chemistry literature. Perhaps I should add some disclaimer. It happens that my name appears in some of the equations in textbooks, the ‘Marcus’ equation for electron transfer or the RRKM theory of gas phase reactions, so an impartial observer might detect some bias on this matter, and that is true.

There is much that has been and is done with these many equations in theoretical chemistry. They provide experimentalists and theoreticians with a hands-on feel of a phenomenon, sometimes suggesting new correlations within and outside the field, and typically providing cohesiveness and a generic quality to many experimental observations. In my own experience I have seen these interactions and results many times in the electron transfer and gas phase reaction fields.

On the other hand, there is a limit to what the equations can do even in the most skilful hands. The field of chemical reaction rates, both in the gas phase and in solution, provides an example. A cornerstone of the theory of the rates of chemical reactions, transition state theory, is due in large measure in the 1930s to Henry Eyring and to M. G. Evans and Michael Polanyi, with insightful input by others, particularly Eugene Wigner. Here, if one knows the details of the interaction of the reacting molecules until they pass through a critical step, a ‘point of no return’, the ‘transition state’, and then applies the statistical mechanics of Gibbs or of quantum descendents of Gibbs, one can calculate the rate constant *k* of the reaction and how it changes with temperature—one can calculate the activation energy *E*_a_ and the pre-exponential factor *A* in the famous late 19th century Arrhenius equation, 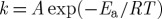, an equation still in force today. In the 1930s, 1940s and 1950s and even 1960s, the *a priori* calculation of an activation energy *E*_a_ was crude. The *a priori* calculation of a pre-exponential factor *A* to an order of magnitude or so gave ballpark estimates that were useful.

The revolution since then in much of theoretical chemistry has been that introduced by electronic computations. One can now calculate the interaction of the reactants in a chemical reaction with much more accuracy than before, though often short of what is desired. There have been many innovations and developments in applications, ranging from simple reactions to reactions in complex biological systems, with their ionic or other channels, conformational changes, electron, proton, hydride or other transfers, and folding and unfolding of proteins. Computations are playing a major role in treating these systems, although the computations themselves of course ultimately use the Schrödinger, Newton and statistical mechanical equations.

There are many positive consequences of these computational developments, particularly for treating systems in a more accurate fashion, and they can yield new insights. There are, of course, limits as to what one can do. Many fluctuations or conformational changes in biological systems occur in the millisecond to second time range while the time range of modern molecular dynamics computer simulations is six to nine orders of magnitude shorter in time. In such cases one may resort to indirect methods such as applying transition state-type theory, or some more sophisticated equivalent, to the individual steps in some overall process.

There is also, in some theorists’ view, a negative aspect to this revolution: now, with the assistance of a computer everybody can be a theoretician! To some extent that is true, but the quality of the product of a computation is usually no better than the quality of its input. One can generate a considerable computer output with little insight before and after the event. The picture is an evolving one and it is too early to write an ending, but it will be interesting to see the insights that these computations will provide.

Computations can also be regarded as a form of ‘laboratory’ experiment, an experiment close to or far from reality, depending on the validity of the input. Indeed, I can recall several examples, one being the long time tails observed in statistical mechanical simulations (Alder & Wainwright 1960, 1970) and the explanation in terms of hydrodynamics.

Another example in my own immediate experience occurred in 1965 when interpreting recent classical and quantum mechanical computational results that appeared in the literature. The results were for the simplest reaction treated computationally, the transfer of an H atom from a H_2_ molecule to another H atom in a linear arrangement. I saw that I could interpret these numerical computational results in the literature, on the effect of vibrational energy on the probability of reaction, by introducing a concept that I termed ‘vibrational adiabaticity’. In this concept a vibration remained in the same quantum state, or in the classical case retained the same value of the vibrational action variable, throughout the course of the reaction, even though the nature of that vibration changed enormously (Marcus 1965*a*). The concept had been briefly mentioned some 30 or so years before, unbeknownst to me, but both the classical and the quantum computational results provided the first direct confirmation. While the term itself has become widely used, it was the computer results that prompted it. In later analytical work in 1966 I obtained corrections for non-adiabaticity. We can well imagine more elaborate and broadly applicable concepts that will emerge in this computation century.

We turn next to the single molecule studies of fluorescing nanoparticles, which will illustrate some of these points. Most of the discussion will focus on a certain class of these particles, but the goal has a broader aim. In a sense single molecule studies although now widespread are still in an early stage. Even for the most widely studied system, CdSe nanoparticles, new facts are being discovered while we write. Not only is the theoretical picture incomplete but also the experimental one on which competing theories are based. What we learn from studies of this particular group of systems can in some measure provide a base not only for suggesting new experiments but also for developing theoretical ideas on new systems in the single molecule field as a whole.

## 3. Nanoparticles and colloid chemistry

Colloid chemistry is well recognized as a venerable well-studied field. Since the colloidal sizes range from micrometres to nanometres, the study of ensembles of nanoparticles is a branch of colloid chemistry. They are correspondingly ubiquitous. Colloidal gold nanoparticles, for example, gave us the millennium-old ruby stained glass, ruby because of the light reflected by the gold clusters.

Inorganic semiconductor nanoparticles, such as quantum dots (QDs), have been widely used in biological imaging. Their high spectral absorption and narrow fluorescence spectrum are attractive features. The QDs are small enough that the absorption and fluorescence spectrum depends upon the radius of the QD, the smaller the QD the higher the energy needed to excite the electron, in accordance with the usual ‘particle in a box’ equation, and similarly the shorter the wavelength of its fluorescence. This dependence of the fluorescence wavelength on the QD size is part of its attraction for use in imaging.

**Figure 1.**
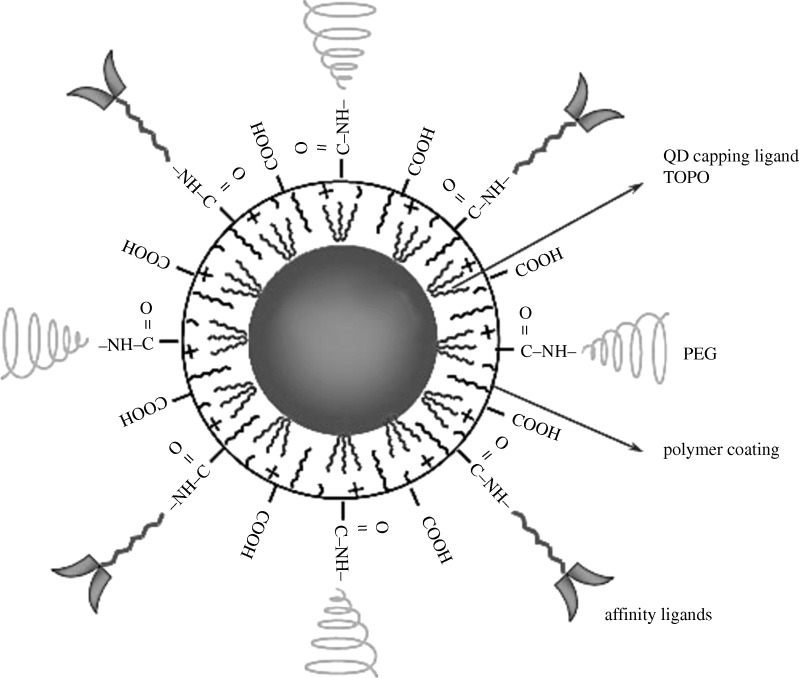
CdSe quantum dot with ZnS coating and various attachments for sensing. Adapted from Gao *et al*. (2004). Reprinted with permission.

Different biological tags can be put on different size QDs, as in figure 1, and many different biological entities can then be sensed independently. Their stability, compared with organic dyes, especially when protected by some coating is another attractive feature of these QDs. Each semiconductor nanoparticle is typically encapsulated in a thin layer of another semiconductor that has a larger band gap and so prevents a photoexcited electron or hole in the core semiconductor from escaping too far. An example is given in figure 1 (Gao *et al.* 2004). The latter also shows the protective coating TOPO (trioctylphosphine oxide) to protect against aggregation. It shows that there are tags, widely used in biological sensing and imaging experiments, but not in the experiments considered below.

## 4. Fluorescent nanoparticles at the single molecule level

For the past 20 years the fluorescence from nanocrystals has been intensely studied in ensembles and increasingly also at the single molecule level. The use of this technique for studying many other systems, particularly biological systems, is now widespread. Ensembles of nanoparticles may have both static and dynamic heterogeneity. In ensembles in which the fluorescence decay shows deviations from mono-exponential behaviour, and perhaps also those that do not, single molecule spectroscopy may help in determining the dynamics on a more detailed level and provide insight into the ensemble behaviour. In any event information is learned that is usually not accessible in ensemble studies. One question that is sometimes asked is whether these single molecule techniques displace the previous ensemble techniques. For obtaining a complete set of data, we found, single molecule and ensemble data are complementary sources. Trajectories that contribute to the ensemble measurements frequently take such a long time that the corresponding single molecule signal is too small to be studied. We discuss this complementary nature of the two types of experiments in §5.

The particular aspect that I would like to focus on for these nanoparticles is their intermittent fluorescence that occurs when some of them are illuminated. We will consider primarily an inorganic system and cite a few examples of organic ones.

**Figure 2.**
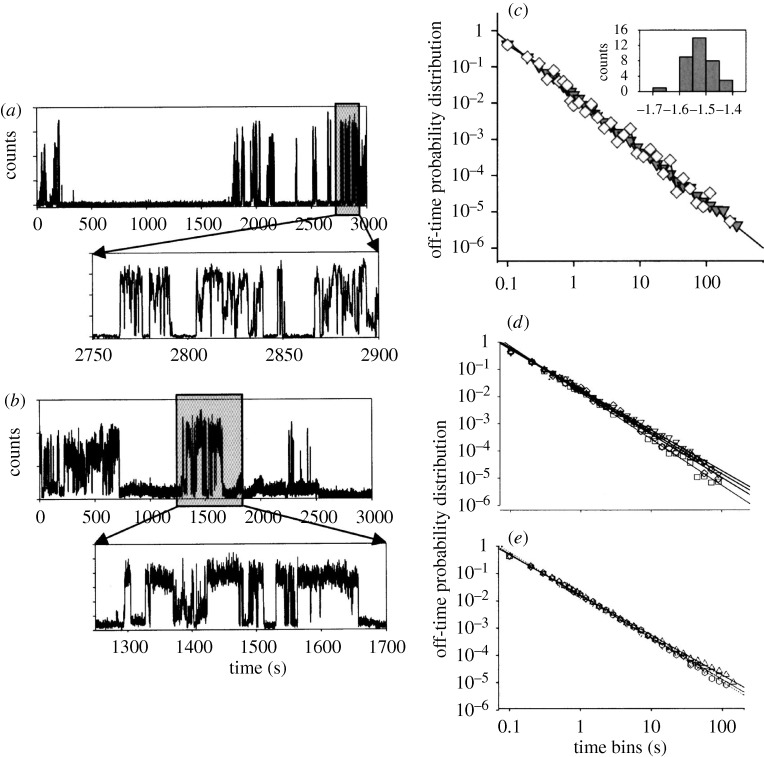
Fluorescence intermittency distribution of ‘off’ times for CdSe(ZnS) QD showing intermittency at (*a*) room temperature and (*b*) 10 K. Self-similarity is seen in the expanded view. (*c*) Normalized off-time probability distribution for different QDs, the straight line having a slope of −1.5 and the inset showing the distribution of powers in this power law; (*d*) off-time probability distribution for different temperatures of QDs; (*e*) off-time probability distribution for different QD radii at room temperature (Shimizu *et al.* 2001). Reprinted with permission.

Since the 1990s there have been many studies of the intermittent fluorescence of semiconductor nanoparticles. Recent reviews include Efros & Rosen (2000), Cichos *et al*. (2007) and Frantsuzov *et al*. (2008). Examples are CdSe, InP and CdTe. They are usually the so-called II–VI and III–V semiconductors, in deference to the chemical groups that the ions belong to in the Periodic Table. When such a nanoparticle is illuminated it fluoresces. Under this continuous illumination it suddenly stops fluorescing, and then with the illumination still continuing it suddenly fluoresces again, and so on, as seen in figure 2 (Shimizu *et al*. 2001). Something new has happened and something new has been learned.

**Figure 3.**
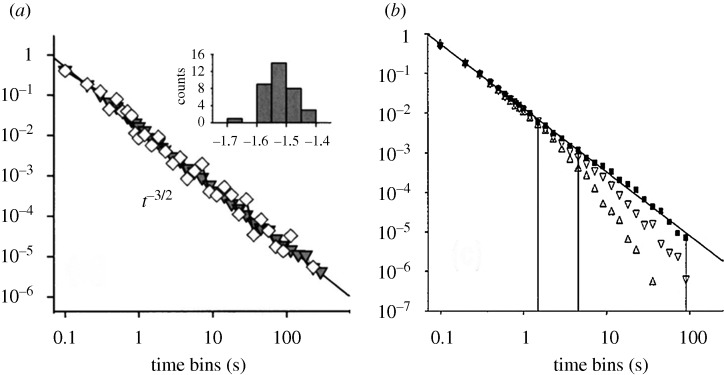
Power law plot slope for ‘on’ and ‘off’ distributions. The distributions are shown for two different temperatures and two different incident intensities. (*a*) Off-time distribution. (*b*) On-time distribution. Black square, 10 K, 175 W cm^−2^; inverted triangle, increased laser power; triangle, increased temperature (Shimizu *et al.* 2001). Reprinted with permission.

In this particular case of fluorescence the distribution of times *P*(*t*) when the nanoparticle goes from ‘on’ to ‘off’ in time (*t*+d*t*) divided by d*t*, or vice versa, i.e. the so-called waiting time distribution for ‘on’ to ‘off’ *P*_on_(*t*) or ‘off’ to ‘on’ *P*_off_(*t*), does not follow an exponential decay with time and so is not a simple decay process. Instead, these *P*(*t*)s follow a power law. A plot of log *P*(*t*) versus 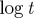 is frequently linear for these nanoparticles, sometimes with an exponential cut-off. Examples are given in figures 2 and 3 (Shimizu *et al*. 2001). A central question is: what is the origin of this power law behaviour and how can one explain a value of the slope of this log–log plot that is often near −1.5, as well as other properties to be discussed? Though most of these results have been observed for inorganic semiconductor nanoparticles, a similar behaviour of a power law has been seen for organic nanoparticles or small molecules as well, e.g. Hoogenboom *et al*. (2007). An early report on single molecule fluorescence intermittency in organic or biochemical systems is given in Moerner & Orrit (1999).

There are two or three alternative theories that have been proposed to explain the single molecule results. A key aspect, addressed by each, is why do these fluorescing particles cease fluorescing for a while and then resume, for both the organic and the inorganic nanoparticles. If a fluorescing organic molecule undergoes an electron transfer and becomes an ion, because of an electron ejection to another molecule or to another plot of the system, the ion is often non-fluorescent and so the molecule will have become ‘off’. It will fluoresce again if and when the electron returns to the organic molecular ion. This intermittency is observed in a single molecule study. For example, if the electron leaves a photoexcited dye molecule that is absorbed on a TiO_2_ nanoparticle (the first part of a common solar energy conversion device), the fluorescing dye becomes ‘off’, and then becomes ‘on’ again if the electron returns to it, yielding fluctuations observed in a recent single molecule study (Wu *et al*. 2009).

More commonly in semiconductor nanoparticles undergoing continuous illumination, CdSe is a common example, an electron or a hole may escape from the main body of the QD to its surface (the theory in this paper) or to some environment outside (other theories). The residual QD is now charged. Efros & Rosen (1997) pointed out that there is now an extra particle in the body of the QD and that condition opens up routes for a fast radiationless decay of an exciton after photoexcitation, and so the QD has become ‘off’ (dark). This new radiationless process that is competitive with the fluorescence, new to QDs but old to atomic physics, is an example of the Auger effect. We depict this Auger process later.

**Figure 4.**
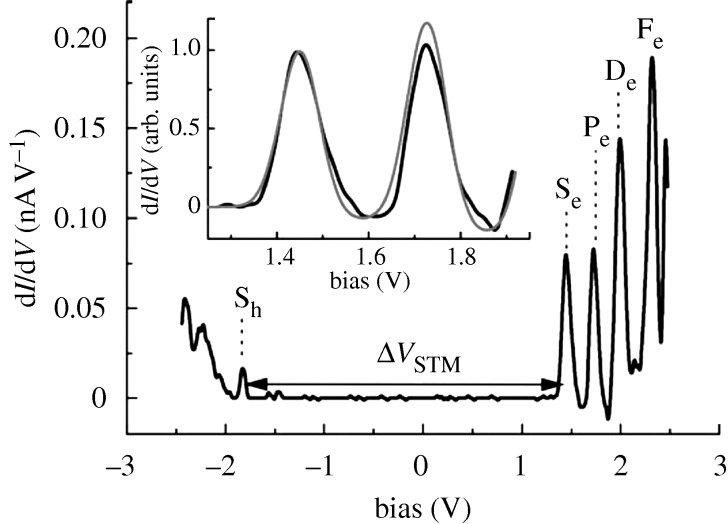
Tunnelling spectroscopy of a CdSe nanocrystal, showing the tunnelling resonances corresponding to the discrete energy levels of the QD (Liljeroth *et al*. 2006). Reprinted with permission.

These QDs have discrete energy levels rather than the conduction and valence bands of the bulk semiconductors. An example is seen in a scanning tunnelling microscopy (STM) plot in figure 4 (Liljeroth *et al*. 2006). The levels there, S_e_,P_e_,D_e_,…, would in the bulk semiconductor be part of the conduction band continuum, while levels such as S_h_… would be part of the valence band. For brevity, we continue to use this band terminology.

The interface between the core of the QD and an outer thin shell of a coating (frequently ZnS when the core is CdSe) typically has a mismatch in the lattice spacing, as well as, one expects, other defects. It can thus act as a trap for a newly photoexcited electron or hole. Indeed, there appear to be traps in a QD over a wide range of time scales. In the case of a CdSe QD, one possible such trap arises from a Se^2−^ ion at the CdSe/ZnS interface, a ‘dangling’ Se^2−^ ion. Unlike a Se^2−^ in the bulk of a QD, it is not completely surrounded by Cd^2+^ ions that fit neatly into a lattice. It is thereby less stable towards losing an electron to any newly created hole in the QD's valence band when the QD is photoexcited.

**Figure 5.**
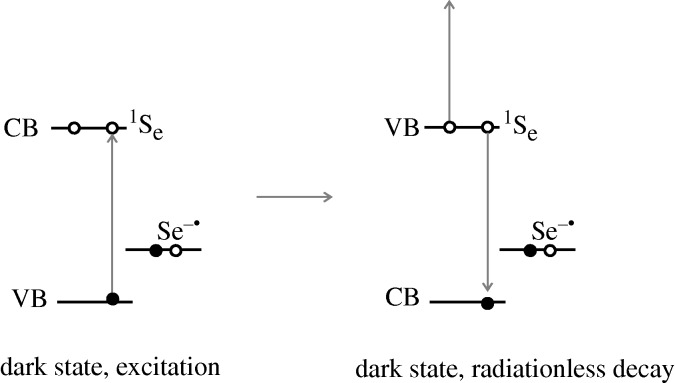
The QD is ‘off’ because the indicated Auger transition can occur when a trap is occupied. Here, the occupied trap is in the form of a Se^−^ in a site formerly occupied by a dangling Se^2−^ at the surface of the QD. The Se^2−^ has trapped a hole. Open circle, electron; filled circle, hole.

Before considering the trapping and detrapping processes we first recall the explanation (Efros & Rosen 1997) as to why a trapped state in the QD causes it to be ‘off’. We do so using the specific model just presented, and depicted in figure 5. It is seen there how for an ‘off’ state the photoexcitation of a second electron from the valence band to the conduction band results in a situation where there is now a radiationless alternative to the fluorescence. There are many electronic states of the QD where the second excited electron can go while the other electron in the conduction band goes into the newly created hole in the valence band in this radiationless transition. Since the QD is now ‘off’ one would infer, in this interpretation, that the Auger process for the radiationless transition is much faster than the competitive fluorescing under these conditions.

**Figure 6.**
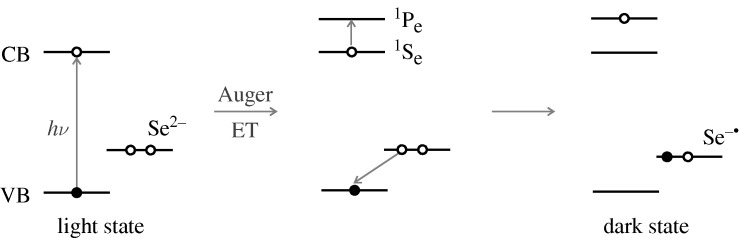
Auger-based trapping mechanism, converting here a dangling Se^2−^ ion to a Se^−^, with the extra electron now occupying a state S_e_ in the conduction band. Open circle, electron; filled circle, hole.

A key question is how does the trapping transition occur? In a QD there are discrete energy levels in the ‘conduction band’ and in the valence band, though the latter are more closely spaced. The STM result in figure 4 is consistent with this model. When the energy difference of the two lowest energy levels in the ‘conduction’ band, S_e_ and P_e_, in figure 6 matches the energy change there for the transfer of an electron from dangling Se^2−^ ion to the newly created hole in the state S_h_ in the ‘valence band’ in figure 6 then a transition may occur and then the QD becomes ‘off’. This ‘resonance’ is between these two states of the system, the state specified by (1e in S_e_, 1 hole in S_h_, Se^2−^) and another state (1e in S_e_, 0 hole in S_h_, Se^−^). The transition can be written as
4.1
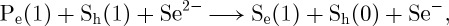

where (1) and (0) denote the number of electrons or holes in the specified state. An analogous transition, without specifying the nature of the surface states, was postulated by Frantsuzov & Marcus (2005).

**Figure 7.**
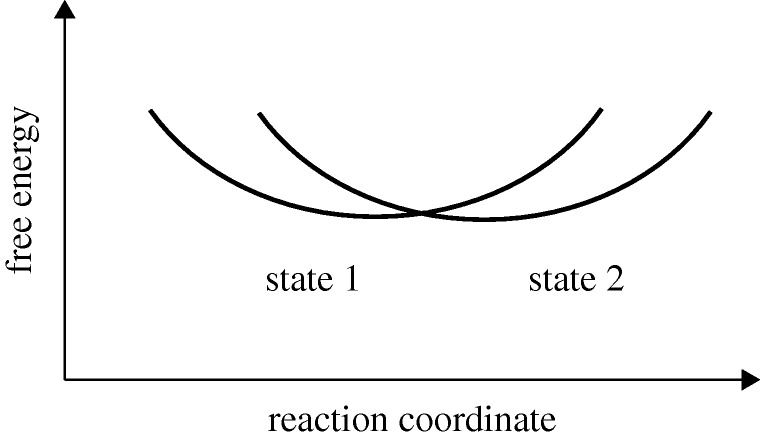
Free energy curves for the two states that are in resonance at the intersection. An example of the two states is given in equation (4.1).

For a discussion of the transitions it is useful to introduce, as in figure 7, free energy curves. These curves are, as in electron transfer reactions (Marcus 1960, 1965*b*), the result of analysing the intersection of two potential curves in a many-dimensional coordinate space. A generalized reaction coordinate (the energy difference of the two energy surfaces at each point) was introduced and the problem was reduced by a statistical mechanical averaging to a discussion in terms of free energy curves.

When the above resonance occurs the system has reached by diffusion the intersection of the two free energy curves in figure 7. This diffusion in energy space may involve small fluctuations in the structure and hence in the ionic charge distribution in the QD. There is direct evidence of fluctuations in energy of the QD. Spectral diffusion is well known from the time-dependent fluctuations in the fluorescence frequency of the QD (Neuhauser *et al.* 2000). Here, it is a fluctuation in the energy difference between an electron being in the valence state and it being in the conduction band, the states that are involved in fluorescence.

The problem to be solved now for this trapping or detrapping is that of treating two electronic states of an entire system that diffuses in energy space. To this end we solve a differential equation, a reaction–diffusion equation, for motion on the free energy curves, and in which there is a transition between the two states. When the system reaches the intersection of the two curves in figure 7, there is some probability, a very small probability if the ‘Auger’ electronic coupling is small, that the system will transfer from one curve to the other.

**Figure 8.**
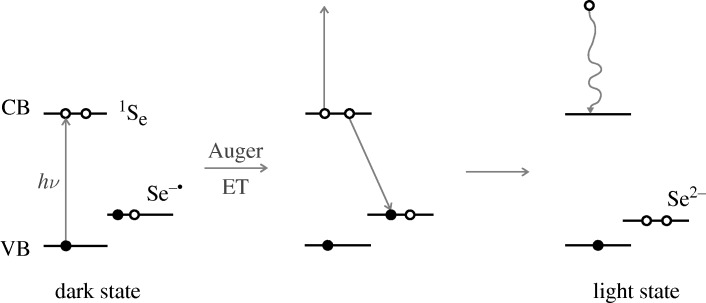
Auger-based mechanism for detrapping, converting a dangling Se^−^ to a dangling Se^2−^. Open circle, electron; filled circle, hole.

How then can this now ‘off’ QD become ‘on’ again? In principle, it could occur if the electron that now exists in the conduction band returns to the trap, the Se^−^ ion, for example by a tunnelling process accompanied by the release of many phonons of the lattice, many phonons because of the large electronic energy difference. However, experiment tells us that the ‘detrapping’ is almost entirely photo-induced rather than spontaneous. The spontaneous return takes an hour or more (Chung & Bawendi 2004). A photo-induced Auger mechanism for detrapping in the photoexcited system is depicted in figure 8.

A differential equation for the probability density along some generalized coordinate *Q* is given by equations (4.2) and (4.3) for the case that we seek the distribution of waiting times for the QD to go from ‘off’ to ‘on’ or vice versa. If *ρ*_1_(*Q*) denotes a probability density (Tang & Marcus 2005*a*):
4.2
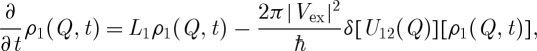

where the diffusion operator is given by
4.3
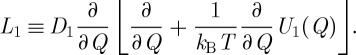

Here, *D*_1_ is a diffusion coefficient in this energy space on curve 1, *U*_1_(*Q*) is the free energy curve for the state 1, *U*_12_ is the energy difference of the two states and the last term in equation (4.2) is a statement that this transition from curve 1 to 2 is a weak transition, and is given by a Fermi's Golden Rule expression.

The diffusion equation was solved for a different model of the trap, a near-band-edge trap (Tang & Marcus 2005*a*,*b*,*c*,) but the mathematical formalism is the same as for the present deep trap Auger-induced model. The differential equation was solved numerically. So here, the computational aspect entered. However, we were also able to obtain an approximate analytic solution that covered all of the time regimes observed in the single molecule experiments. The part that it did not cover was the extremely long times needed to reach a steady state of an ensemble under continuous illumination. Here there was a different analytical solution. Those extremely long times were obtainable only in ensemble experiments since the single molecule fluorescence was too weak to be observed at very long times.

We recall the approximate Laplace transform solution to the reaction–diffusion equation for all but the longest times, yielded (Tang & Marcus 2005*a*)
4.4
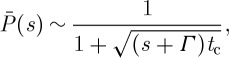

where *t*_c_ and *Γ* are parameters that depend on the quantities appearing in the differential equation.

From the inversion of the equation it was found that the time dependence of this waiting time distribution was (Tang & Marcus 2005*a*,*b*,*c*)
4.5
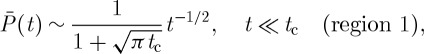

(4.6)
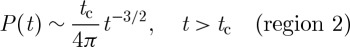

4.7
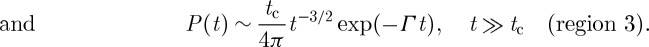

For still longer times equation (4.8) was obtained instead of the above equations:
4.8
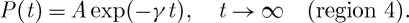

When the diffusion is anomalous, the 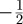 and 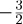 become 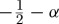 and 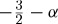, where *α* is approximately 0–0.5 (Tang & Marcus 2005*b*). The critical time *t*_c_ depends upon the reaction rate at the intersection of the two free energy curves and on the diffusion constant for motion on the surface.

The physical origin of the time regimes in equations (4.5)–(4.8) is of interest and we comment on it briefly. In the first of these time regimes represented by equation (4.5) a steady state is building up at the intersection of the two free energy curves in figure 7. During this time the probability population at the intersection begins to approach zero because the intersection serves as a sink for that probability. During this period the survival probability distribution varies as *t*^−1/2^. In the second of these time regimes, a steady state has been established and the lifetime distribution varies as *t*^−3/2^. In somewhat oversimplified terms the survival probability distribution near the sink behaves as a well known (*Dt*)^−1/2^ and the survival probability distribution function (waiting time distribution) is the time-derivative of this function.

In the next time regime for this model, given by equation (4.7), the effect of a finite slope of the curves at the intersection on the diffusion becomes apparent: it causes a ‘forced diffusion’ that enhances the rate of loss from the intersection region and decreases the survival population rate of change exponentially. In this regime, the rate varies as 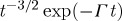. In the final regime, given by equation (4.8), the calculated survival time probability distribution is a pure exponential and is due to escape from the bottom of the free energy curve in which it resides.

**Figure 9.**
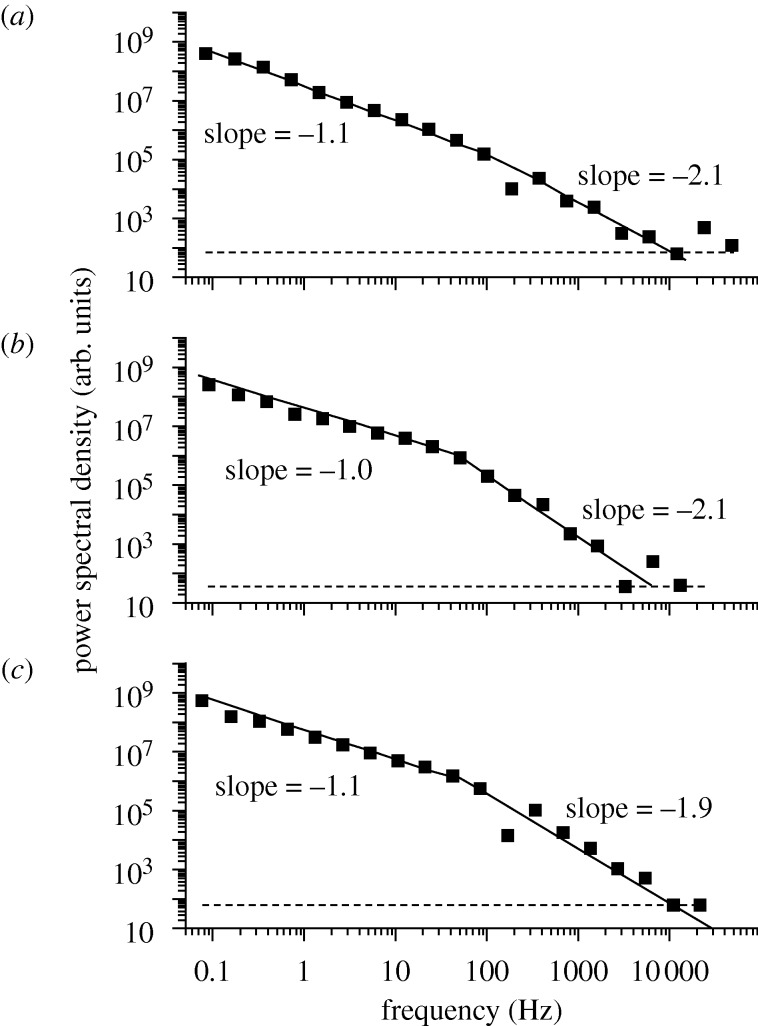
Power spectral density of fluctuations in fluorescence measured for three individual QDs. Solid lines are fitted power laws to low-frequency and high-frequency portions of the power spectra, and horizon dashed lines are expected shot-noise levels (Pelton *et al*. 2007).

Recently, this prediction of a change in slope of a 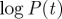 versus 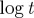 plot seen in equations (4.2) and (4.3) was tested experimentally by studying the power spectral density for the distributed lifetimes of the QDs (Pelton *et al*. 2007). The prediction from equations (4.2) and (4.3) was that there be a change in the slope of unity in this quantity at some time *t*_c_. The prediction of a change in slope, perhaps the only one thus far for QDs, was later confirmed in experiments by Pelton *et al.* (2007). The results are reproduced in figure 9. This confirmation does not mean that the theory is correct, of course, but it is one hurdle that was crossed.

This reaction–diffusion model serves to explain a number of the facts, such as the tendency for the power in the power law to be approximately 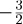 on the average, and there being a cut-off for the ‘on times’, and the prediction of a change in slope. However, how does one explain the asymmetry—why is there not a cut-off for the ‘off’ times, or at least not an easily discernable cut-off in the time scale of intermittency?

An important new set of experiments was performed for different excitation wavelengths at room temperature (Knappenberger *et al.* 2007). The power law for the ‘off’ behaviour was observed at all excitation energies. In the new experiments a pure power law for the ‘on’ behaviour was observed at low excitation energies. At higher energies, sufficient to excite the electron from the valence band to the P_e_ state, there was the often seen exponential cut-‘off’ for the ‘on’ state. Thus far, this energy dependent behaviour has not been adequately explained. So, the following comments on this issue are preliminary and need to be explored further.

When the QD is ‘off’ any further photoexcitation, even to a high energy, is expected to result in a quick relaxation of the newly photoexcited electron to its lowest state in the conduction band, because of an Auger process. In this case, regardless of excitation energy, any subsequent transition always occurs from the lowest state in the conduction band (S_e_). When the QD is ‘on’, any high-energy excitation is expected to be followed by a slower relaxation to the S_e_ state and so there is a greater opportunity for some other transition, such as ‘on’ to ‘off’, to occur. This possibility is absent when the photoexcitation is at low energy. So in this case of an ‘on’ state there can be a difference in the log–log plot, depending on the excitation energy.

There are several additional facts that reflect on the mechanism. The S_e_ to P_e_ transition seen in figure 6 is known from a S_e_→P_e_ infrared absorption spectrum occurring when a hole is deliberately created by coating the surface of the QD with a thiocresol. A photoexcited thiocresol readily loses an electron to the S_e_ state in the conduction band to the QD (Shim & Guyot-Sionnest 2000), and so the S_e_→P_e_ optical transition can be observed. Another relevant observation involves evidence that the trapped hole is localized and diffusing about rather than distributed as a band over the surface of the QD. Evidence of a diffusing charge is seen in the effect of an alternating electric field on the fluorescence of the QD (Park *et al*. 2007). A possible explanation of the effect of both enhancement and decrease of the fluorescence when the field is increasing is that of a trapped charge hopping from site to site on the surface rather by a delocalized band.

## 5. Ensemble versus single molecule studies

To describe the difference between ensemble and single molecule trajectories we can use figure 7. To this end we let state 1 be the ‘on’ state that is reached by photoexcitation of an ‘on’ QD. In an ensemble of trajectories distributed in thermal equilibrium in state 1 most of the QDs will fluoresce, but a few will diffuse thermally to the intersection of the two curves in figure 7. There they will have some probability of undergoing a transition to state 2, the ‘off’ state. At very short times only the most excited QDs in state 1 will have enough energy to reach the intersection, but at longer times systems coming from near the bottom of the well of state 1 will also reach the intersection by thermal fluctuation. Ultimately after some transient period a steady state near the intersection will occur of systems that remain in state 1. There will also be trajectories that entered the ‘off’ state now undergoing the transition from ‘off’ to ‘on’. Eventually in an ensemble experiment there will be some steady-state ratio of ‘on’/‘off’ QDs, about 0.2 in a recent experiment (Chung & Bawendi 2004).

These ensemble trajectories differ in their starting point from those that occur in the single molecule experiments. In the latter, when a system goes from ‘on’ to ‘off’ it starts at the intersection of the two curves in figure 7 and not at the bottom of a well. Most of these trajectories will return to that intersection before reaching the bottom of a well (the fluorescence signal in these single molecule experiments is usually too weak to be observed for that long a time). We see that the trajectories for the single molecule experiments and those from ensemble experiments differ, and are complementary.

## 6. Epilogue

In these many studies of the intermittent fluorescence of CdSe nanoparticles much has been learned about factors influencing their behaviour. We have only given some of the experimental observations. The intermittent fluorescence depends upon many factors, such as the intensity and wavelength of the incident light, size of the nanoparticles, temperature, dielectric environment, electric fields, shape, such as whether they are nanoparticles or nanorods, and the coating, for example, whether it changes gradually or sharply.

The present description is mainly a discussion of some of the issues that arise and by no means an exhaustive survey of the field. Analytic theory can provide a possible base for understanding many experimental observations of QDs. In the example presented here we extended a diffusion controlled electron transfer model (Tang & Marcus 2005*a*; Frantsuzov *et al*. 2008; Marcus 2009), with new data yet to come and to be explained. From the experimental data one obtains values of parameters such as *t*_c_ and *Γ* that appear in the analytical expressions, just as in experiments on the rates of chemical reactions one obtains rate constants from the experimental data. In both cases suitable computations may provide values of parameters deduced from the experiments and then compared with the latter. In the case of QDs computations may use existing electronic structure computations for the QDs, to calculate the various transition probabilities that are involved.

In the present case of QDs there is a complexity involving factors such as different kinds of surface states, other irregularities of the surface, and the extent of electron confinement. The new experiments serve as ‘straitjackets’, forcing the theory to account for an increasingly wide body of data. The evolution of this field for inorganic and organic nanoparticles may eventually make an interesting chapter in texts on chemical reaction rates.

The theory I have focused on is only one approach, a diffusion controlled electron transfer approach that appears to be consistent with the data. It is certainly far from being a final answer on a topic whose experiments are still open-ended and surprising. In my experience approximate models nevertheless have provided a start that later may develop into more general theories. There are other theories that should be discussed. We leave that discussion to another publication, perhaps more timely when the new data on intensity and wavelength effects become available.
